# Nomogram for the Prediction of High-Grade Dysplasia and Invasive Carcinoma in Patients With Intraductal Papillary Mucinous Neoplasms of the Pancreas Based on Variables of Noninvasive Examination

**DOI:** 10.3389/fonc.2021.609187

**Published:** 2021-03-09

**Authors:** Bo Li, Xiaohan Shi, Suizhi Gao, Shuo Shen, Yun Bian, Kai Cao, Yaqi Pan, Guoxiao Zhang, Hui Jiang, Gang Li, Shiwei Guo, Gang Jin

**Affiliations:** ^1^ Department of Hepatobiliary Pancreatic Surgery, Changhai Hospital Affiliated to Navy Medical University, Shanghai, China; ^2^ Department of General Surgery, Beidaihe Rehabilitation and Recuperation Center of PLA, Qinhuangdao, China; ^3^ Department of Radiology, Changhai Hospital Affiliated to Navy Medical University, Shanghai, China; ^4^ Department of Pathology, Changhai Hospital Affiliated to Navy Medical University, Shanghai, China

**Keywords:** intraductal papillary mucinous neoplasms, pancreas, surgery indication, nomogram, noninvasive examination

## Abstract

Intraductal papillary mucinous neoplasms (IPMNs) are a heterogeneous group of neoplasms and represent the most common identifiable precursor lesions of pancreatic cancer. Clinical decision-making of the risk for malignant disease, including high-grade dysplasia and invasive carcinoma, is challenging. Moreover, discordance on the indication for resection exists between the contemporary guidelines. Furthermore, most of the current nomogram models for predicting malignant disease depend on endoscopic ultrasonography to evaluate the precise size of mural nodules. Thus, this study aimed to propose a model to predict malignant disease using variables from a noninvasive examination. We evaluated patients who underwent resection of pathologically confirmed IPMNs between November 2010 and December 2018 and had preoperative clinical data available for review. Based on binary multivariable logistic regression analysis, we devised a nomogram model to predict malignant IPMNs. The area under the receiver operating characteristics curve (AUC) was used to evaluate the discrimination power of the model. Of the 333 patients who underwent resection of IPMNs, 198 (59.5%) had benign and 135 (40.5%) had malignant IPMNs. Multivariable logistic regression analysis showed that cyst size, cyst location, cyst wall enhancement, multicystic lesion, diameter of main pancreatic duct, neutrophil-to-lymphocyte ratio, serum carbohydrate antigen 19-9, and carcinoembryonic antigen were significantly associated with malignancy. The nomogram, constructed based on these variables, showed excellent discrimination power with an AUC of 0.859 (95% CI: 0.818–0.900, *P* < 0.001). In conclusion, we have developed a nomogram consisting of a combination of cross-sectional imaging features and blood markers, variables that can readily be obtained by noninvasive examinations during the surveillance period, which can distinguish benign from malignant IPMNs. Nevertheless, external validation is warranted.

## Introduction

Intraductal papillary mucinous neoplasms (IPMNs) of the pancreas are a heterogeneous group of lesions that grow within the ductal system of the pancreas (1). Interest in IPMNs is growing because of their frequent identification on routine cross-sectional imaging and because they comprise the most common radiographically identifiable precursors of pancreatic carcinoma (2). These cystic lesions are believed to progress from low-grade dysplasia (LGD), to high-grade dysplasia (HGD), and to invasive cancer and may involve the main pancreatic duct (MPD), branch ducts, or both (3). Previous studies have reported a malignancy risk of 38%–68% for main duct IPMNs (MD-IPMNs), 38%–65% for mixed-IPMNs, and 12%–47% for branch duct IPMNs (BD-IPMNs) (4). Thus, the current guidelines suggest resection of MD-IPMNs and mixed-IPMNs (5–7). However, the communication of BD-IPMNs with the MPD may be difficult to establish (4), and radiology seems to both underestimate (for BD-IPMNs) and overestimate (for MD/mixed-IPMNs) the involvement of the MPD in the final pathologic examination (8–10). Moreover, discordance on the indication for surgery of IPMNs exists in the guidelines, especially between the International Consensus Fukuoka Guidelines (revised in 2017) (5), European evidence-based guidelines (revised in 2018) (6), and the guidelines published by the American Gastroenterological Association (7) and the American College of Gastroenterology (11). Hence, current recommendations for routine IPMNs resection have led both to overtreatment and to missed malignancy, including HGD and invasive disease, which in turn poses a considerable challenge among practitioners who are deciding between referring patients for aggressive surgical intervention or conservative surveillance. In addition, patients who undergo aggressive pancreatic surgical intervention bear a substantial risk of postoperative complications and mortality. The risk of malignant degeneration must be balanced against the risks and benefits associated with definitive surgical management, patient comorbidities, and life expectancy (12).

Although nomogram models may be useful in predicting malignant disease, most of the constructed nomogram models depend on preoperative endoscopic ultrasonography (EUS), which could identify the precise size of mural nodules (MNs) (10, 13). However, EUS is not available or convenient in a number of regions in China; thus, we aimed to propose a new nomogram model with a low rate of EUS performance. Moreover, some reports suggest that preoperative neutrophil-to-lymphocyte ratio (NLR) (14) and platelet-to-lymphocyte ratio (PLR) (15) are useful predictive biomarkers for malignant potential in patients with IPMNs or pancreatic cystic neoplasms. Thus, we intended to incorporate these biomarkers to a nomogram model and use them to optimize the existing models. The model was mainly constructed using variables from radiologic and experimental examinations, which could be performed in basic hospitals; thus, it could be easily developed and applied in underdeveloped regions.

## Materials and Methods

### Study Population

Prior to institutional review board approval of this retrospective study, we identified patients with resected IPMNs in our database. We obtained data on 450 consecutive patients with pathologically confirmed IPMNs diagnosed between November 2010 and December 2018 at the Department of Hepatobiliary Pancreatic Surgery, Changhai Hospital. Between 2010 and 2012, patients recommended to undergo surgery included those fulfilling the “Sendai positive” criteria, i.e., with a tumor size ≥3 cm, symptomatic, with MNs or a thickened wall, or with a dilated MPD of ≥6 mm. According to the 2012 and the 2017 revised Fukuoka guidelines, between 2013 and 2018, patients were recommended to undergo upfront surgery if they had “high-risk stigmata”. Patients with worrisome features who were found to have a definite MN ≥5 mm, suspicious MPD involvement, or suspicious cytology during additional EUS examinations were suggested to undergo resection. In this study, patients with worrisome features might have undergone surgical resection without additional EUS examinations after discussing the risks and benefits in Multiple Disciplinary Team (MDT) of pancreatic neoplasms. Patients who did not meet the criteria for resection were monitored according to the contemporary guidelines.

**Figure 1:**
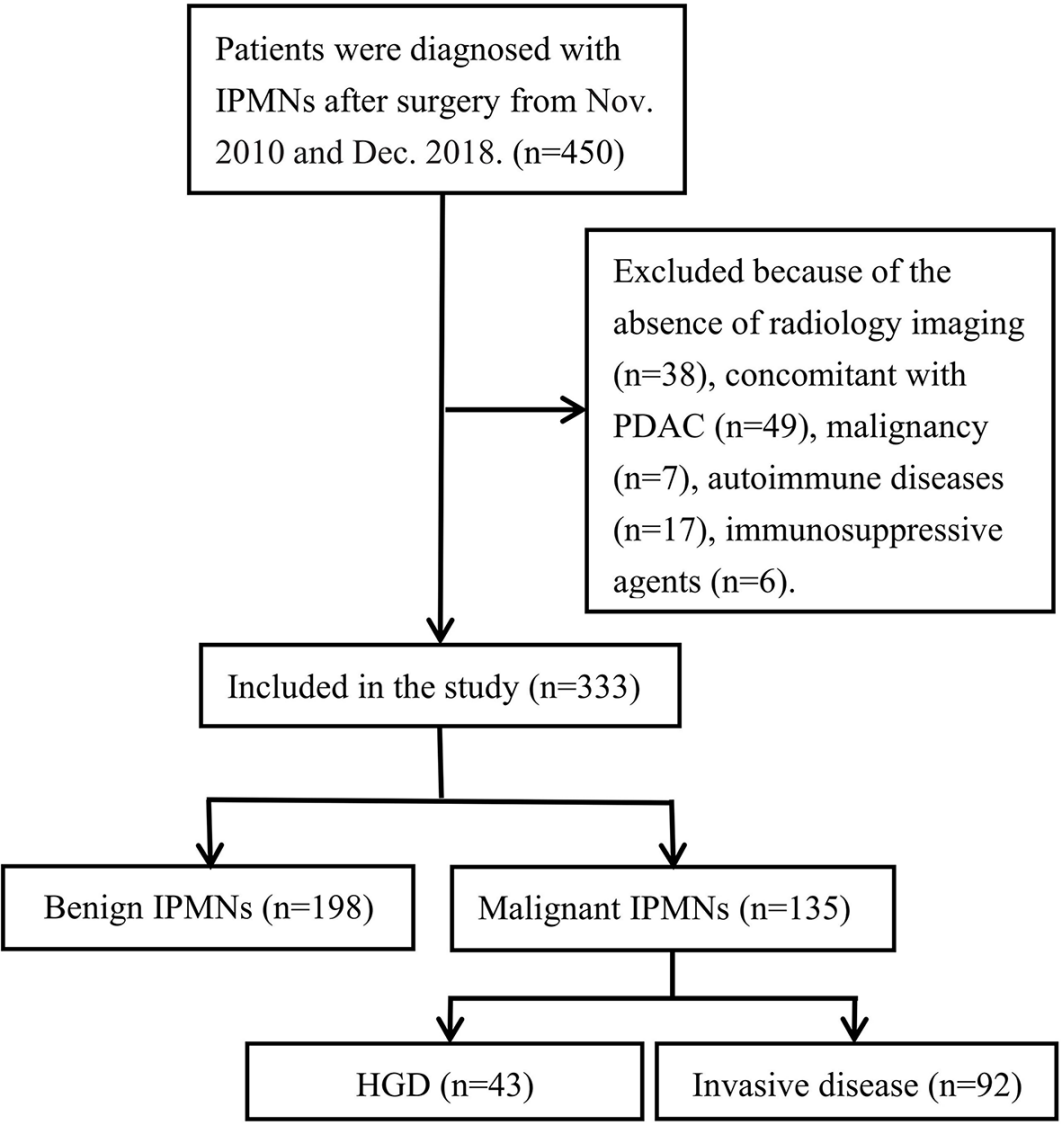
Flowchart of patient selection.

Forty-nine cases for which radiology imaging, including computed tomography (CT) or magnetic resonance imaging (MRI), was not performed preoperatively were excluded. Patients with IPMNs co-occurring with pancreatic ductal adenocarcinoma (PDAC), which were not continuous of IPMNs, and with PDAC notable by microscopical examination, were also excluded (**Figure 1**). Information on demographics (sex and age), and chief complaint (pain, jaundice, pancreatitis, and other symptoms) was retrospectively obtained from the database. Data on tumor markers (carcinoembryonic antigen [CEA], carbohydrate antigen [CA] 19-9) and blood routine test (NLR, PLR) within 1 month prior to surgery were also collected. To secure cohort homogeneity regarding preoperative values of neutrophil, platelet, and lymphocyte counts, further exclusion criteria were applied: personal history of prior, synchronous, or metachronous malignancy; transplantation; autoimmune disease; human immunodeficiency virus infection; and treatment using immunosuppressive agents (**Figure 1**).

A blinded gastrointestinal radiologist reviewed the preoperative imaging and recorded cyst characteristics. The following imaging features were assessed: maximal diameter of the dominant cyst, cyst location in the pancreas (i.e., head/uncinate/neck, body and tail, and multifocal), cyst wall enhancement, presence of a solid component in the cyst, multicystic lesion, multiple lesions, presence and enhancement of a MN (defined as a mural-based soft tissue component projecting into the cyst), maximal diameter of the MPD (i.e., <5, 5–9, and ≥10 mm), and lesion type (i.e., BD-IPMNs, MD-IPMNs, and mixed-IPMNs).

### Statistical Analysis

The risk of malignant disease was the outcome of interest. The nomogram was built based on a stepwise multivariate binary logistic regression model. Continuous data were transformed into categorical data by the median value or the cut-off value with the maximum Youden index. Categorical variables are presented as percentages. All variables associated with the outcome with a significance level of *P* < 0.05 on univariate analysis were candidates for the multivariate analysis. Only the variables that remained as significant predictors of the outcome in multivariate regression analysis were included in the final model. A receiver operating characteristics (ROC) curve was used to measure the predictive accuracy on each testing data set. Area under the ROC curve (AUC) was employed to express how well the model could discriminate between patients with malignant and those with benign disease. Higher values indicate better discrimination; a value of 0.5 indicates no predictive discrimination, whereas 1.0 indicates perfect separation. The diagnostic potential for different cut-off values of malignancy probability in the nomogram were evaluated by calculating the sensitivity, specificity, positive predictive value (PPV), negative predictive value (NPV) and accuracy based on the true positive, false positive, true negative, and false negative for malignancy diagnosis. Statistical analyses were performed using R software version 2.13.2 and SPSS version 25 (SPSS, Chicago, IL). Statistical significance was defined as two-tailed *P* < 0.05.

## Results

### Patient Characteristics

**Table 1** summarizes the clinicopathologic and demographic characteristic of the patients. Of the 333 patients who underwent IPMN resection, 135 (40.5%) had a malignant disease, including 43 patients with HGD and 92 patients with invasive carcinoma, and 198 (59.5%) had LGD (benign disease). The male-to-female ratio was 1.71 (210:123). The age ranged from 19 to 83 years (median 63 years). Approximately 2/3 of the lesions were located in the pancreatic head/uncinate/neck. BD-IPMNs were the most frequently resected lesions. The median cyst size and diameter of MPD were 2.6 cm and 8 mm, respectively, and the median values of pre-pancreatectomy PLR and NLR were 157.58 and 2.66, respectively. CT, MRI, and EUS were performed in 94.0%, 70.3%, and 38.1% of the patients, respectively.

**Table 1:** Characteristics of patients with IPMN who underwent pancreatic resection.

**Total number of patients**	333
**Age at pancreatectomy** (years), range (median)	19–83 (63)
**Sex ratio,** M:F	210:123
**Tumor location** (Head/uncinate/neck):(body/tail):multifocal, n	221:97:15
**Type of lesion** BD : MD:mixed, n	164:141:28
**Cyst size** (cm), range (median)	0.2–22 (2.6)
**MPD diameter** (mm), range (median)	2.9–50 (8)
**PLR**, range (median)	38.82–1,307.14 (157.58)
**NLR**, range (median)	0.37–50.79 (2.66)
**Pathological diagnosis** LGD : HGD:invasive disease, n	198:43:92
**CT** performed, n (%)	313 (94.0%)
**MRI** performed, n (%)	234 (70.3%)
**EUS** performed, n (%)	127 (38.1%)

BD, branch duct; CT, computed tomography; EUS, endoscopic ultrasonography; HGD, high-grade dysplasia; IPMN, intraductal papillary mucinous neoplasms; LGD, low-grade dysplasia; MD, main duct; MPD, main pancreatic duct; MRI, magnetic resonance imaging; NLR, neutrophil-to-lymphocyte ratio; PLR, platelet-to-lymphocyte ratio.

### Univariate and Multivariate Analysis

Among the variables tested, 13 were independently associated with malignant IPMNs (**Table 2**). Tumor located in the pancreatic head/uncinate/neck, cyst wall enhancement, cyst with a solid component, CEA ≥5 ng/ml, serum CA 19-9 ≥37 u/ml, MPD diameter ≥10 mm, cyst size ≥3 cm, NLR ≥2, and PLR ≥120 were strongly associated with malignancy (*P* < 0.001). Multivariate analysis (based on the binary logistic regression) of factors identified as significant in the univariate analysis showed that cyst wall enhancement (*P* = 0.001), serum CEA ≥5 ng/ml (*P* = 0.047), serum CA 19-9 ≥37 u/ml (*P* < 0.001), MPD diameter ≥10 mm (*P* = 0.029), cyst size ≥3 cm (*P* = 0.006), and NLR ≥2 (*P* = 0.005) were independent risk factors for malignant IPMN. Moreover, tumor located in the body/tail (*P* = 0.021) and multicystic lesion (*P* = 0.033) were independent protective factors for malignant IPMNs (**Table 2**).

**Table 2:** Univariate and multivariate analyses of risk factors for malignant IPMNs.

	Benign, n (%)	Malignant, n (%)		Univariate OR (95% CI)	*P*	multivariate OR (95% CI)	*P*
**Total**	198 (59.5)	135 (40.5)				
**Sex,** male	121 (61.1)	89 (65.9)		0.812(0.515,1.282)	0.371	–	
**Age,** >60 years	111 (56.1)	84 (62.2)		1.291(0.826,2.019))9)	0.262	–	
**Chief complaint**							
Other symptoms	101 (51.0)	68 (50.4)		**ref**		**-**	
Pain	79 (39.9)	47 (34.8)		0.884(0.550,1.420)	0.609	–	
Jaundice	4 (2.0)	13 (9.6)		4.827(1.510,15.43)	**0.008**	–	
Pancreatitis	14 (7.1)	7 (5.2)		0.743(0.285,1.936)	0.543	–	
**Cyst location**							
Head/uncinate/neck	123 (62.1)	98 (72.6)		**ref**		**ref**	
Body/tail	71 (35.9)	26 (19.3)		0.460(0.273,0.774)	0.004	0.455 (0.233,0.890)	**0.021**
Multifocal	4 (2.0)	11 (8.1)		3.452(1.066,11.17)	0.039	2.773 (0.637,12.06)	0.174
**Cyst size,** >3 cm	68 (34.3)	82 (60.7)		2.958(1.880,4.654)	**<0.001**	2.259 (1.264,4.038)	**0.006**
**Cyst wall enhancement,**	33 (16.7)	56 (41.5)		3.544(2.135,5.884)	**<0.001**	3.188 (1.637,6.211)	**0.001**
**Solid component of cyst,** present	4 (2.0)	16 (11.9)		6.521(2.129,19.97)	**0.001**	**-**	
**Enhancing mural **						**-**	
Without	176 (88.9)	106 (78.5)		**ref**		–	
With & <5 mm	18 (9.1)	21 (15.6)		1.937(0.987,3.801)	0.055	–	
With & ≥5 mm	4 (2.0)	8 (5.9)		3.321(0.976,11.30)	0.055	–	
**Multicystic lesion,** present	47 (23.7)	17 (12.6)		0.463(0.253,0.847)	**0.013**	0.417 (0.187,0.931)	**0.033**
**Multiple lesions,**	6 (3.0)	5 (3.7)		1.231(0.368,4.117)	0.736	–	
**MPD**							
<5 mm	28 (14.1)	11 (8.1)		**ref**		**ref**	
5–9 mm	95 (48.0)	46 (34.1)		1.233(0.564,2.692)	0.600	1.638 (0.628,4.274)	0.313
≥10 mm	75 (37.9)	78 (57.8)		2.647(1.231,5.695)	0.013	2.872 (1.111,7.421)	**0.029**
**IPMN type**							
BD	111 (56.1)	53 (39.3)		**ref**		–	
MD	70 (35.4)	71 (52.6)		2.124(1.355,3.381)	0.001	–	
Mixed	17 (8.6)	11 (8.1)		1.355(0.593,3.095)	0.471	–	
**CEA serum,** ≥5	20 (10.1)	43 (31.9)		4.160(2.312,7.483)	**<0.001**	2.242 (1.011,4.972)	**0.047**
**CA19-9 serum,** ≥37 IU/ml	20 (10.1)	82 (60.7)		13.77(7.733,24.52)	**<0.001**	9.102 (4.763,17.40)	**<0.001**
**NLR,** ≥2	107 (54.0)	110 (81.5)		3.742(2.233,6.272)	**<0.001**	2.487 (1.310,4.722)	**0.005**
**PLR, ≥**120	103 (52.0)	100 (74.1)		2.635(1.638,4.239)	**<0.001**	**-**	

BD, branch duct; CA, carbohydrate antigen; CEA, carcinoembryonic antigen; CI, confidence interval; IPMN, intraductal papillary mucinous neoplasms; MD, main duct; MPD, main pancreatic duct; NLR, neutrophil-to-lymphocyte ratio; OR, odds ratio; PLR, platelet-to-lymphocyte ratio. The bold value means significant difference (P<0.05).

### Nomogram for Predicting Malignant Intraductal Papillary Mucinous Neoplasms

**Figure 2:**
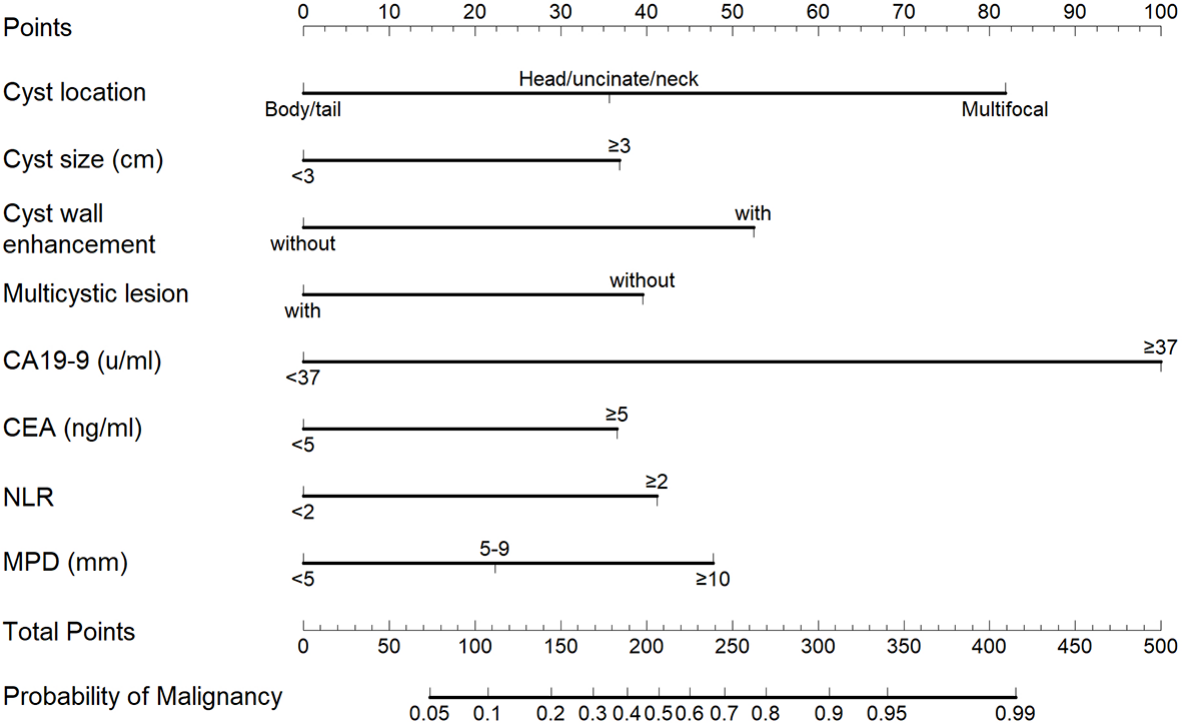
Nomogram for the detection of malignant intraductal papillary mucinous neoplasms. The points on the scale were added for each variable. The total points projected on the bottom scales indicate the probability of malignancy.

**Figure 3:**
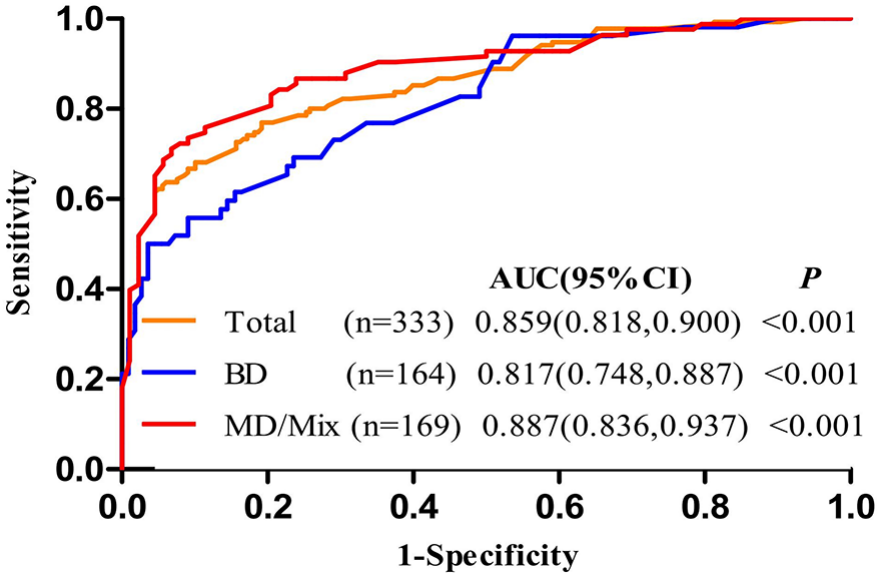
Receiver operating characteristic curve of the nomogram for predicting the probability of malignant intraductal papillary mucinous neoplasms.

**Figure 4:**
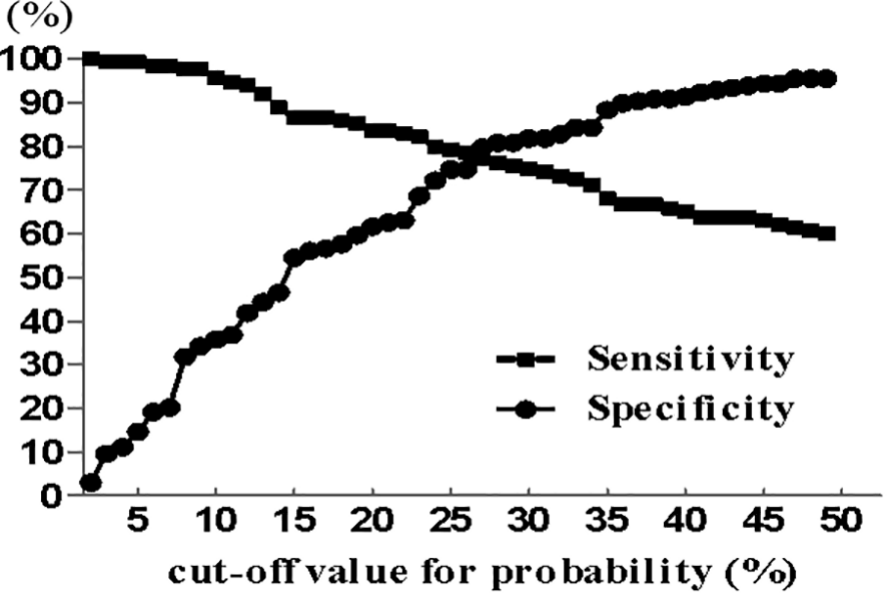
Sensitivity and specificity were estimated based on the data set (n = 333) as functions of the cut-off point for the predicted probability of malignant intraductal papillary mucinous neoplasms.

The nomogram that was generated *via* the binary multivariate logistic regression model, including all significant independent risk factors for malignant IPMNs, is shown in **Figure 2**. To use the nomogram, points on a scale of 0 to 100 are assigned to each predictor and the sum is the final score (“total points” axis with a vertical ruler); the ruler is followed down to read predicted malignancy probability. **Figure 3** shows the AUC for the prediction of malignancy (0.859, 95% confidence interval [CI] 0.818–0.900). Based on the relationship of sensitivity, specificity, and malignancy probability shown in **Figure 4** and using this nomogram, in patients with ≥20% predicted probability of malignancy who underwent surgery, the nomogram could detect malignant IPMNs (sensitivity) in 86.7% (117/135), thereby sparing 43.4% (86/198) of the patients without malignancy from undergoing an unnecessary surgical procedure (1-specificity). When the predicted probability of malignancy was set at ≥30% and ≥40%, the sensitivity decreased, and the specificity and the accuracy increased compared with that of ≥20% (**Table 3**). The PPV and NPV of the nomogram were 73.5% (100/136) and 82.6% (162/196), respectively, when the probability of malignancy was set at ≥40%. Meanwhile, under this circumstance, the accuracy was the highest among the three probabilities of malignancy shown in **Table 3**.

**Table 3:** Diagnostic potential for different cut-off values of malignancy probability in nomogram.

Malignancy probability	TP	FN	FP	TN	PPV	NPV	SEN	SPE	Accuracy
**Total**									
**≥20%**	117	18	86	112	0.58	0.86	0.87	0.57	0.69
**≥30%**	108	27	55	143	0.66	0.84	0.80	0.72	0.75
**≥40%**	101	34	36	162	0.74	0.83	0.75	0.82	0.79
**BD**									
**≥20%**	41	12	38	73	0.52	0.86	0.77	0.66	0.70
**≥30%**	36	17	28	83	0.56	0.83	0.68	0.75	0.73
**≥40%**	31	22	16	95	0.66	0.81	0.58	0.86	0.77
**MD/Mix**									
**≥20%**	76	6	48	39	0.61	0.87	0.93	0.45	0.68
**≥30%**	72	10	27	60	0.73	0.86	0.88	0.69	0.78
**≥40%**	70	12	20	67	0.78	0.85	0.85	0.77	0.81

FN, false negative; FP, false positive; IPMN, intraductal papillary mucinous neoplasms; NPV, negative predictive value; PPV, positive predictive value, SEN, sensitivity; SPE, specificity; TN, true negative; TP, true positive. Accuracy=(TP+TN)/(TP+TN+FP+FN).

In addition, **Figure 3** shows the AUC for the prediction of malignancy in MD/mixed-IPMNs and BD-IPMNs (0.887, 95% CI 0.86–0.945 and 0.817, 95% CI 0.748–0.887, respectively). Among those with ≥20% predicted probability of pancreatic carcinoma who underwent surgery, the nomogram could predict carcinoma in 169 patients with MD/mixed-IPMNs, with a 92.7% (76/82) sensitivity, 44.8% (39/87) specificity, 61.3% (76/124) PPV, and 86.6% (39/45) NPV. When the predicted probability of pancreatic carcinoma who underwent surgery was set at ≥40%, in the 164 patients with BD-IPMNs, the nomogram had a 58.4% (31/53) sensitivity, 85.6% (95/111) specificity, 65.9% (31/47) PPV, and 81.2% (95/117) NPV (**Table 3**).

## Discussion

Resection of precursor lesions, such as IPMNs, to prevent cancer could be an important method to improve the dismal prognosis of patients with pancreatic cancer. However, risk–benefit outcomes of pancreatectomy with respect to perioperative morbidity and mortality and loss of endocrine and exocrine function would require a precise selection of patients whose IPMNs are at high risk of malignant transformation, considering the relatively dormant nature of most IPMNs, especially BD-IPMNs. Therefore, it is important to identify individuals who will benefit from an early operation and those who will benefit from a watchful waiting approach.

Although contemporary guidelines suggest resection of BD-IPMNs with high-risk stigmata and MD/mixed-IPMNs, discrepancies between radiologic and pathologic evaluations exist in the diagnoses of the type of IPMNs, which in turn lead to unnecessary resection and to missed malignancy in clinical practice. To overcome this limitation, several nomograms or scoring systems to predict malignancy in IPMNs have been proposed (13, 16–18). However, the variables in most of the models include MNs, which are mainly detected by EUS during surveillance of IPMNs (19). In the present study performed at a high-volume center in China, EUS was performed in a low percent (38.1%) of patients with IPMNs of the pancreas who underwent surgery. The low rate of EUS performance may be due to the unavailability of EUS in basic hospitals and the lack of recognition among doctors of the importance of EUS for the diagnosis and management of IPMNs. In addition, the papilla or nodule detection rate by EUS is higher than that by CT and MRI (19, 20), and cross-sectional imaging is more prone to variability in assessment of the presence or absence of MNs (21). However, several studies have reported that presence of a MN was one of the most important predictive factors for malignant IPMN (22–24). In our study, although the rate of MNs detection in patients with malignancy was significantly higher than that in patients with benign lesions, the MNs were not retained in the final multivariable malignancy prediction model, which is consistent with a previous predictive model (25). Furthermore, in one study it was reported that one-third of IPMN-derived carcinomas develop without a MN (26). Moreover, recent guidelines have favored less invasive imaging both during the initial evaluation and during surveillance, and recommend stopping surveillance for those without positive features on cross-sectional imaging over a 5-year period (27). A less invasive examination is associated with high compliance from the patients, especially for long-term surveillance, and results could be obtained easily. Thus, we intended to propose a nomogram model that was mainly based on a less invasive or noninvasive examination, which could be performed in basic hospitals.

All multivariate parameters identified in our study were considered in our predictive model, which included five parameters obtained from cross-sectional imaging (cyst location, cyst size, cyst wall enhancement, MPD diameter, and multicystic lesion) and three from experimental examination (serum CA19-9 and CEA levels, and NLR). Among these variables, cyst size, cyst wall enhancement, MPD diameter, and serum CA19-9 level are included among the risk factors predictive of malignancy or are among the indications for surgery listed in the current guidelines (7). Notably, there is discordance between the International Consensus Fukuoka Guidelines and the European evidence-based guidelines in terms of the cyst size (3 vs. 4 cm) that is predictive of malignancy. In the present study, a cyst size of 3 cm yielded the best predictive accuracy.

Regarding the cyst location, previous studies have shown that head/uncinate cysts are more likely to harbor malignancy compared to cysts located in the neck/body/tail (28). Moreover, pancreatic head cysts or tumors are a predictive factor for malignancy in IPMNs (13, 29–31). In alignment with these findings, we demonstrated that a tumor located in the head/uncinate/neck could significantly predict malignant IPMNs.

Moreover, this is the first constructed nomogram model for predicting malignant IPMNs that includes multicystic lesion. IPMNs with a multicystic lesion were less likely to harbor malignancy, which could be attributed to the grape-like clusters of cysts or multicystic lesions; such clusters are mostly seen in BD-IPMNs (32, 33), which have a relatively low malignancy rate. Another reason may be that solid carcinoma derived from IPMN lesions replaced the primary multicystic lesions (26). Nonetheless, a large cross-sectional imaging study is warranted to validate our findings.

Several studies have indicated that serum CEA could independently predict malignancy in patients with pancreatic IPMNs (13, 34, 35). A meta-analysis also suggested that a serum CEA level ≥5 ng/ml is a good predictor of malignancy and invasiveness. Serum CEA has a low sensitivity and a high specificity for HGD and invasive IPMNs (36); however, its sensitivity may be increased when it is combined with serum CA 19-9 (37). Thus, It is thus not surprising that both serum CEA and CA 19-9 levels were retained in our final nomogram model.

NLR, which is a parameter of systemic inflammatory and immune reactions, may play an important role in the progression of solid tumors (38), including PDAC (39, 40). Recent studies have reported that elevated preoperative NLR is a significant marker for predicting malignant IPMNs (14, 25, 41, 42). Moreover, a previous study revealed that the predictive power for malignant IPMNs is further increased when the host-derived marker of NLR and the tumor-derived markers of CEA and CA 19-9 (41) are combined. Our model, which has a high predictive power, included all three of these markers. Future studies should identify the best combination with host- and tumor-derived markers for predicting malignant IPMNs.

Compared with other nomogram models (10, 13, 18, 43, 44), our model has a relatively higher predictive power for predicting malignancy in BD-IPMNs, MD/mixed-IPMNs, and both. Most of the previous nomograms included MNs, and some included sex, age, symptoms, and weight loss (43, 44). The variables in the present nomogram were ranked according to the OR value as follows: serum CA19-9 ≥37 u/ml, cyst wall enhancement, MPD diameter ≥10 mm, NLR ≥2, serum CEA ≥5 ng/ml, cyst size ≥3 cm, cyst in the body/tail, and multicystic lesion. It is clear that NLR and serum CEA level, which are not included in the latest revised guidelines (5, 6), are essential in our model. Nevertheless, large and intensive research should be conducted to prove such significance.

The present nomogram showed good performance for malignancy prediction compared with the primary nomograms (13, 18, 25). For the overall population regardless of IPMN type, the present study indicated that elder patients with malignancy probability ≥40% should undergo surgery to avoid overtreatment, younger patients with malignancy probability ≥20% should undergo surgery to avoid missed malignancy, and other patients with malignancy probability ≥30% should undergo surgery to balance the risk–benefit outcomes of pancreatectomy. Besides, our research also revealed that MD/mixed-IPMNs patients should undergo surgery when the malignancy probability is ≥20% to avoid missed malignancy, and so should BD-IPMNs patients with malignancy probability ≥40% in order to avoid overtreatment.

This study has notable strengths. The variables used in the nomogram were all based on a noninvasive examination, which indicates that our nomogram model is both reliable and easy to be performed, even by patients in regions where EUS for surveillance is not available.

Nonetheless, several limitations exist in our study. The EUS performance rate was lower in our study than in other studies; thus, the presence of MNs was not detected in the early stage. However, a recent study indicated that the presence of a MN during the initial 5-year surveillance period is associated with a 12-fold increased risk of IPMN-derived carcinoma and that a MN is an indication for surgical resection (26). In addition, MNs were not included in our model; thus, patients at a high risk should be recommended to undergo EUS, with or without fine needle aspiration, to detect malignancy in the early stage, especially younger patients with a high life expectancy and older patients who may not be able tolerate major surgery or anesthesia (19). Hence, external validation for our nomogram model needs to be performed in the future. Moreover, this study was based on retrospective data of patients who underwent surgery in a single institution. Further prospective validation studies based on preoperative cohorts and patients under surveillance from different institutions are needed to confirm the clinical value of our nomogram model.

In conclusion, a combination of cross-sectional imaging features and blood markers were incorporated into a nomogram model to predict malignancy. Our nomogram model was able to distinguish between benign and malignant IPMNs, with the advantage of using noninvasive examination only during the surveillance. Although further studies are required, including external validation, our nomogram model could be a valuable tool in estimating the risk of malignancy in individuals with IPMNs.

## Data Availability Statement

The raw data supporting the conclusions of this article will be made available by the authors, without undue reservation.

## Ethics Statement

The studies involving human participants were reviewed and approved by the Shanghai Changhai Hospital Ethics Committee. The patients/participants provided their written informed consent to participate in this study.

## Author Contributions

BL, SGu, and GJ conceptualized the study idea and design. BL, XS, SGa, SS, YB, KC, YP, GZ, HJ, and GL acquired, analyzed, or interpreted the data. BL, XS, and SGa made the initial drafts. SGu and GJ critically revised the article for important intellectual content. All authors contributed to the article and approved the submitted version.

## Funding

This work was supported by the National Natural Science Foundation of China (grant number: 81672830), the Constructing Project of Clinical Medical Centers (Pancreatic Disease) in Shanghai (grant number: 2017ZZ01009), the “234 Discipline Climbing Plan” Project of the First Affiliated Hospital of Naval Military Medical University (grant number: 2019YXK033), and the Western Medicine Guidance Project of Shanghai Science and Technology Commission (grant number: 16411967200).

## Conflict of Interest

The authors declare that the research was conducted in the absence of any commercial or financial relationships that could be construed as a potential conflict of interest.
